# Supplementary material for: TMEM16F and dynamins control expansive plasma membrane reservoirs
Source: Nat Commun. 2021 Aug 17;12:4990. doi: 10.1038/s41467-021-25286-z (PMC8371123; doi:10.1038/s41467-021-25286-z)
Supplement: Supplementary file 1 — Supplementary Information [file 41467_2021_25286_MOESM1_ESM.pdf]

Supplemental Figures

Supplemental Figure 1

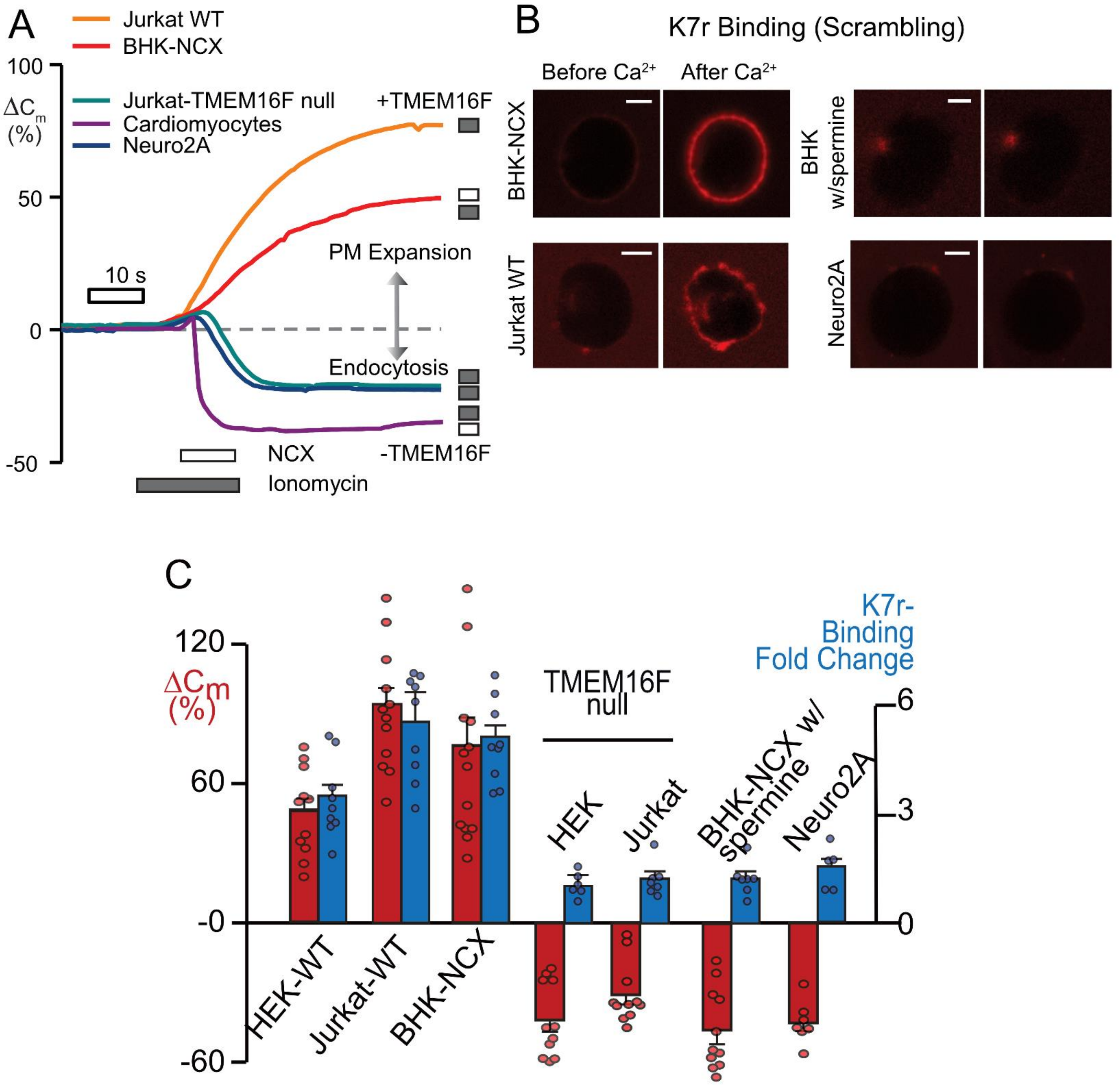

Supplementary Figure 1. A) As in Figure 1A for HEK WT and HEK-TMEM16F-null cells, representative recordings of PM area changes (%ΔC_m) for multiple cell types with or without TMEM16F. All recordings at 37°C, unless stated otherwise. Cells expressing endogenous TMEM16F expand while cells lacking endogenous TMEM16F, ablated through CRISPR or blocked by spermine fail to expand during Ca elevations. B) Cells that expand also scramble PM phospholipids, as in Figure 1A, confocal images showing extracellular binding of K7r. Cytoplasmic spermine (1 mM) used throughout to block TMEM16F activity in BHK-NCX cells. Neuro2A cells lack endogenous TMEM16F. Jurkat TMEM16F-null cells previously reported show no K7r binding after Ca influx via ionomycin (Bricogne et. al., *Scientific Reports*, 2019). C) Composite results for A) B) and Figure 1A for multiple cell lines – K7r binding is fold increase of initial localized PM fluorescence after PM expansion as determined by cross-sectional line scans. For %ΔC_m n=14, 14,14,11,11,11,7,14 and for K7r n=9,9,9,7,7,7,5. All data was analyzed from the total number of independent cells (n) from a minimum of three experiments and expressed as mean ± s.e.m. Unpaired Student's *t*-test was used for comparing two groups. Scale bar: 5 µm.

Supplemental Figure 2

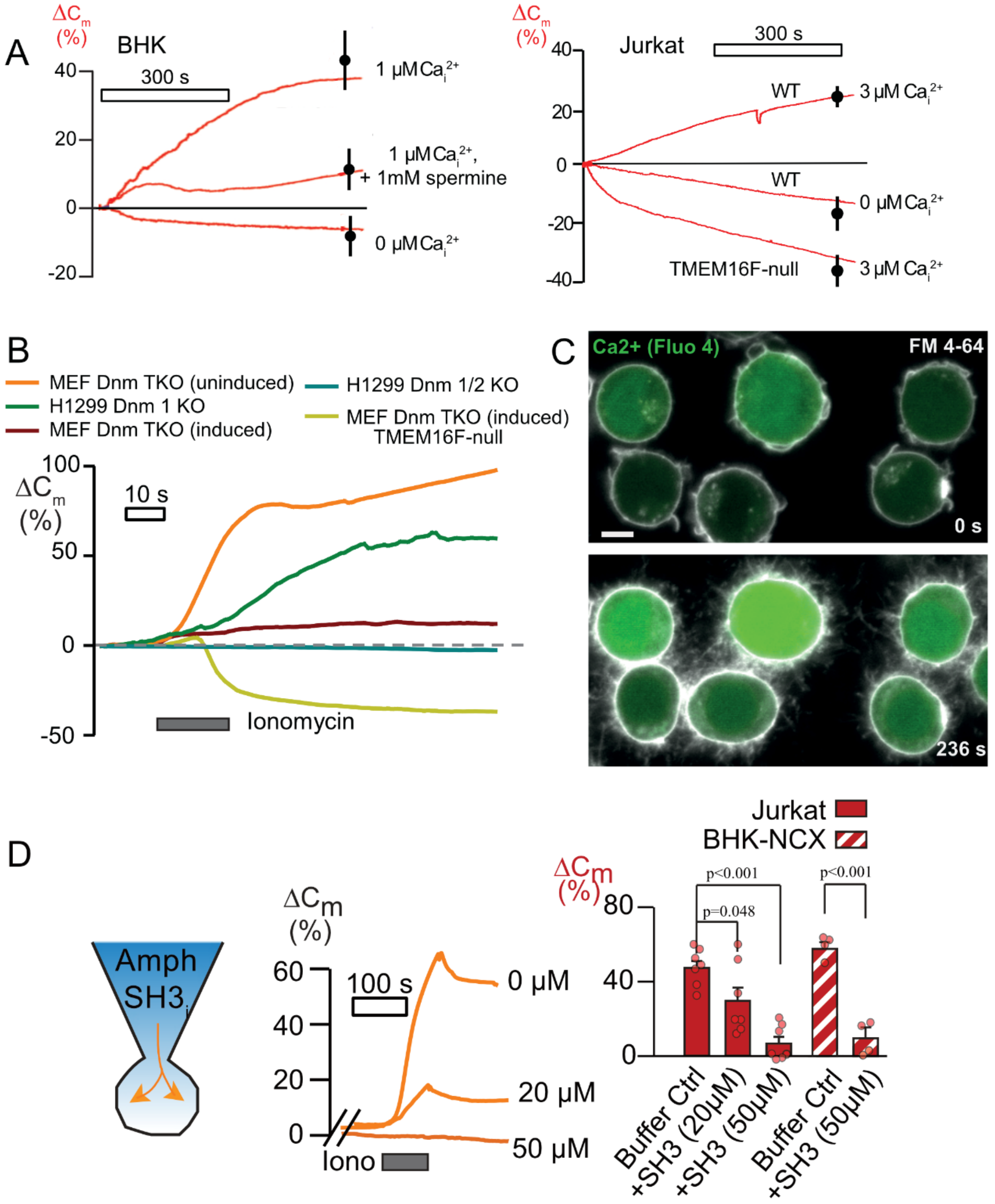

Supplementary Figure 2. A) Intracellular dialysis of lower free Ca concentrations induces PM expansion over several minutes. Expansion is blocked by cytoplasmic spermine (BHK, left), knock-out of TMEM16F (Jurkat, right) and the absence of Ca, n=6. B) Representative capacitance recordings illustrating expansion using Ca-ionomycin (2 mM,10 μM) in Dnm expressing MEF Dnm TKO (uninduced) and H1299 Dnm1 KO cells. With loss of expansion in induced Dnm TKO cells and H1299 cells with additional Dnm2 CRISPR KO. C) An example of prolonged ionomycin (10 μM) exposure (bottom) and extensive membrane shedding similar to Supp. Vid. 1 with Fluo-4 AM (green) to monitor cytoplasmic Ca and the membrane probe FM 4-64 (3 μM, white), instead of K7r, in Jurkat WT cells. For subsequent protocols in this paper, ionomycin or Ca exposure is transient with minimal membrane shedding over the course of the experiments. D) Acute blockade of Ca-activated PM expansion using a dynamin binding domain. In Jurkat and BHK-NCX cells, cytoplasmic dialysis of purified dynamin-sequestering Amphiphysin2 SH3 domains or buffer controls for 300 s prior to activation of TMEM16F with ionomycin or reverse NCX exchanger activation reduced PM expansion in a dose dependent manner, n=7,7,7,4,4 independent cells from two experiments and expressed as mean ± s.e.m. Unpaired Student's *t*-test used for comparing two groups.

Supplemental Figure 3

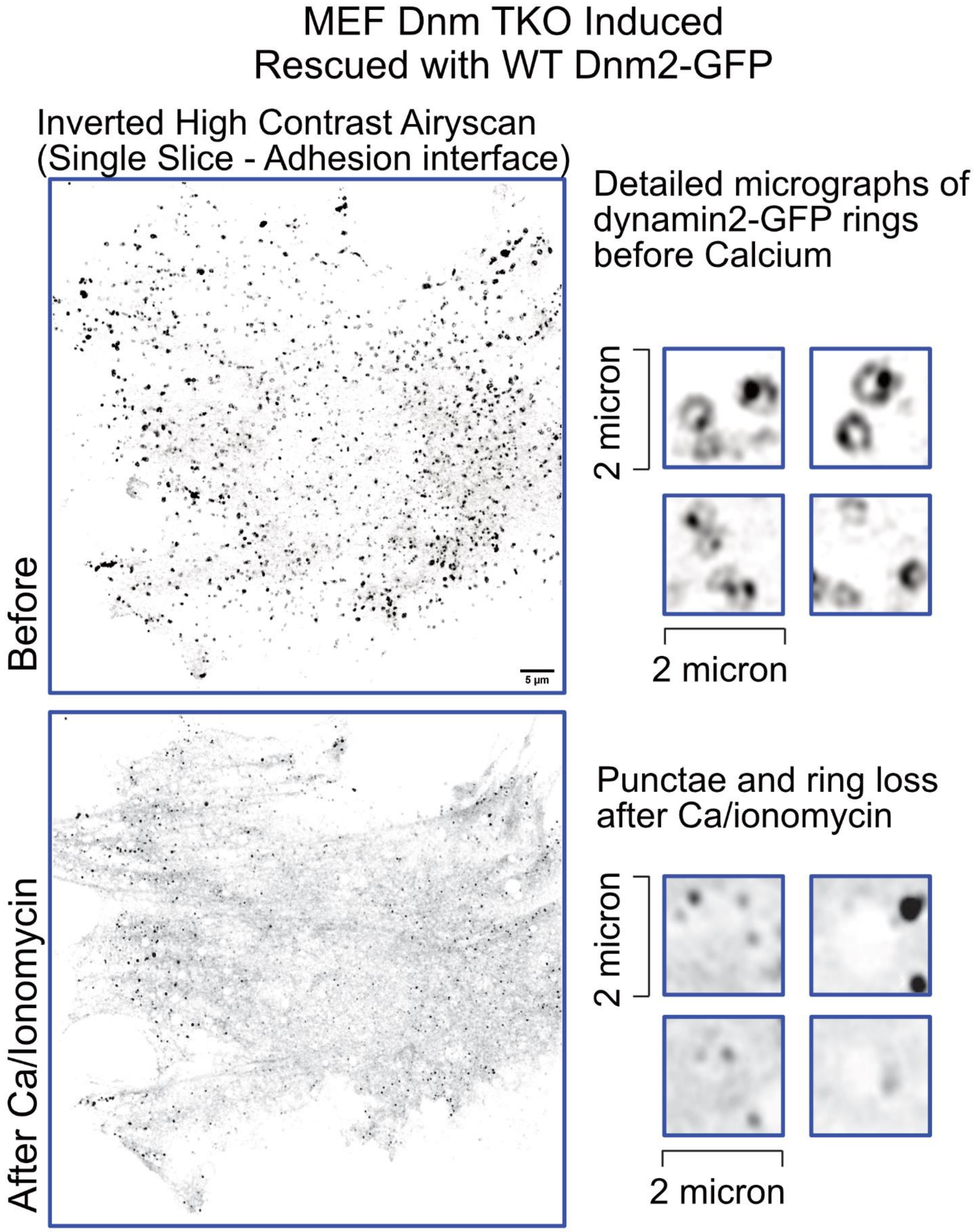

Supplemental Figure 3. Induced (tamoxifen) Dnm TKO cells lacking all three dynamins were rescued with transient expression of WT Dnm2-GFP. Representative experiment of live cell super-resolution Airyscan images reveal that Dnm2 expression appears as punctae clearly visible at the membrane surface near the glass adhesion interface. Detailed 2-micron (X-Y) pictographs reveal that many of the punctae demonstrate ring like constriction points near the cell surface with diameters measured in the hundreds of nanometers or less. After Ca-ionomycin treatment (bottom), as shown in Figure 1B and 1F, Dnm2 disassociates from the membrane interface, increasing bulk cytoplasmic fluorescence and reduces the presence of punctae and ring like formations, n=6 independent cells from 6 independent experiments. Scale bar: 5 µm, inset 4 μm^2.

Supplemental Figure 4

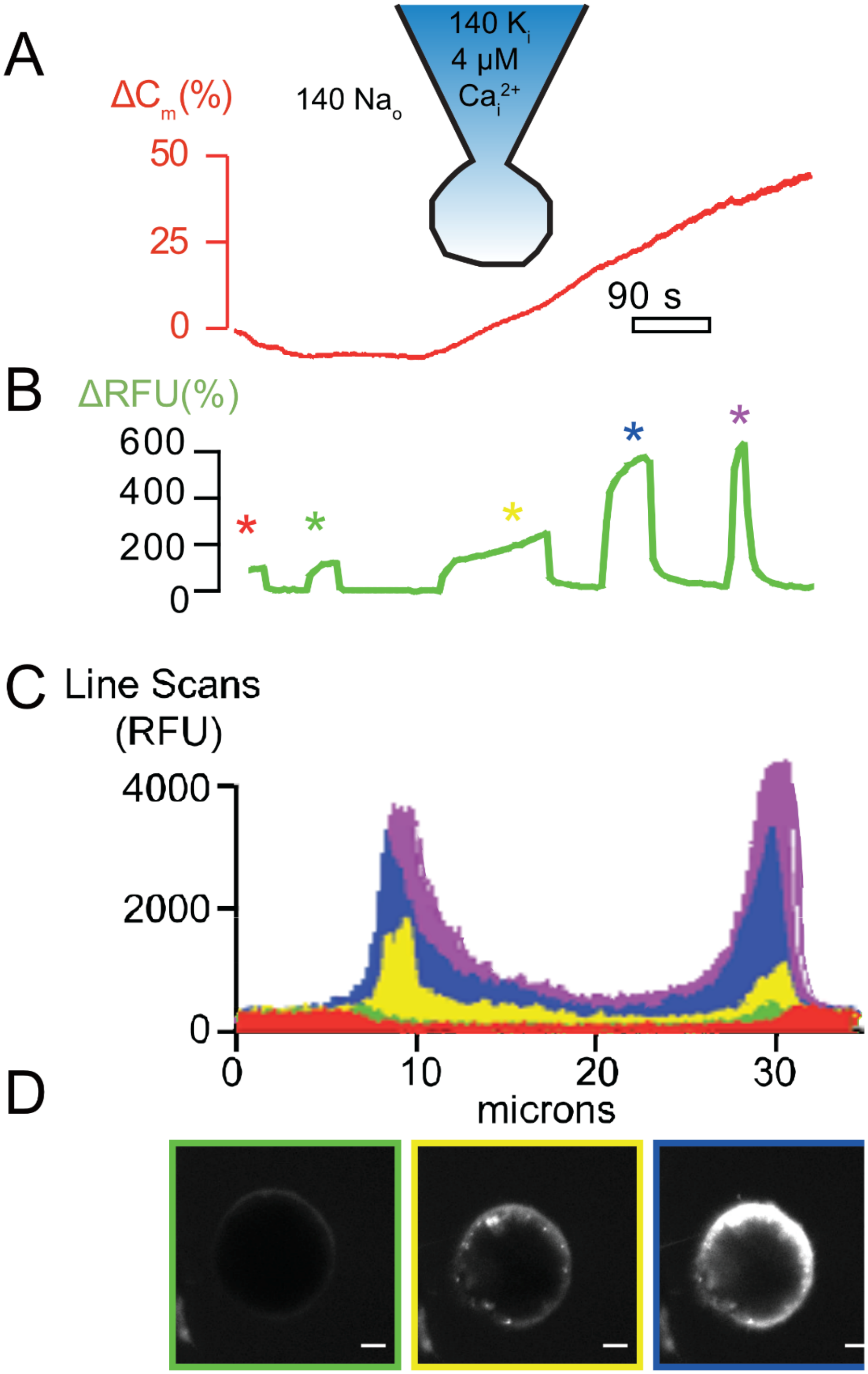

Supplemental Figure 4. PM expansion (A) and exposure of anionic PLs ((B); K7r binding) have similar time courses. Representative example of the time course of membrane scrambling during whole-cell patch clamp recordings of BHK cells. Cytoplasmic pipette solution contained highly buffered Ca (4 µM) that induced expansion slowly over the course of 5 min (see also Sup. Fig. 1C). Repeated exposure of reversibly labeled K7r to the extracellular side revealed scrambling occurs concurrently to with PM expansion and is detected in the newly expanded PM reservoir compartments. The new compartments are exposed to the extracellular side as K7r binding is reversible and the depth of labeling extends into the cytoplasm as revealed by line scan analysis (C/D), n=3. Scale bar; 5 µm

Supplemental Figure 5.

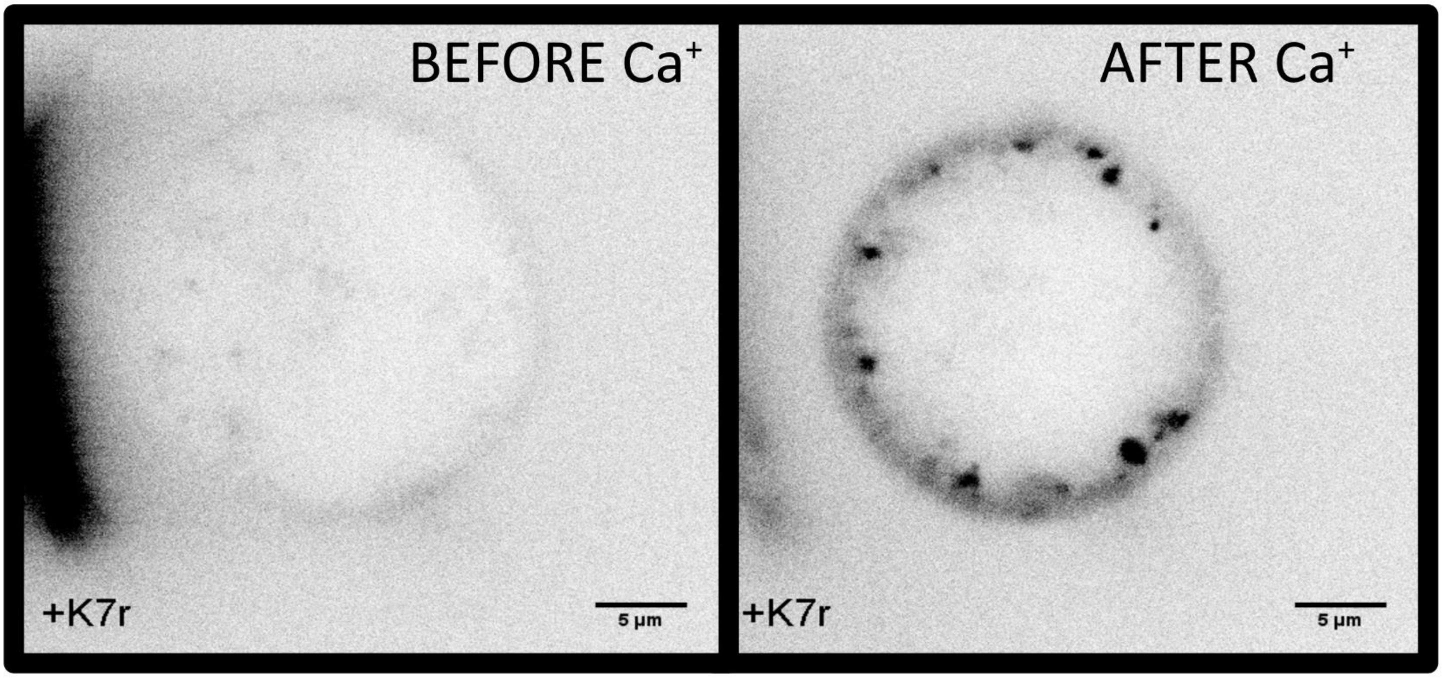

Supplemental Figure 5. Similar experiment to Supplemental Figure 4 with reversible K7r labeling of BHK cells dialyzed with highly buffered Ca (4 µM). As previously published (Bricogne et. al., *Scientific Reports*, 2019), TMEM16F mediated expansion can still occur without Na or K albeit at a slower rate (See also Supp. Fig. 1). Here, the large monovalent cation tetraethylammonium (TEA) replaced intracellular K, and Cs was substituted for Na. Expansion still occurs within 5 minutes and continues for an additional 10 min with reversible labeling with K7r. Deep compartments become exposed to the extracellular space indicating that the new membrane that is revealed during expansion does not appear like canonical vesicular fusion, n=6. Scale bar; 5 µm.

Supplemental Figure 6

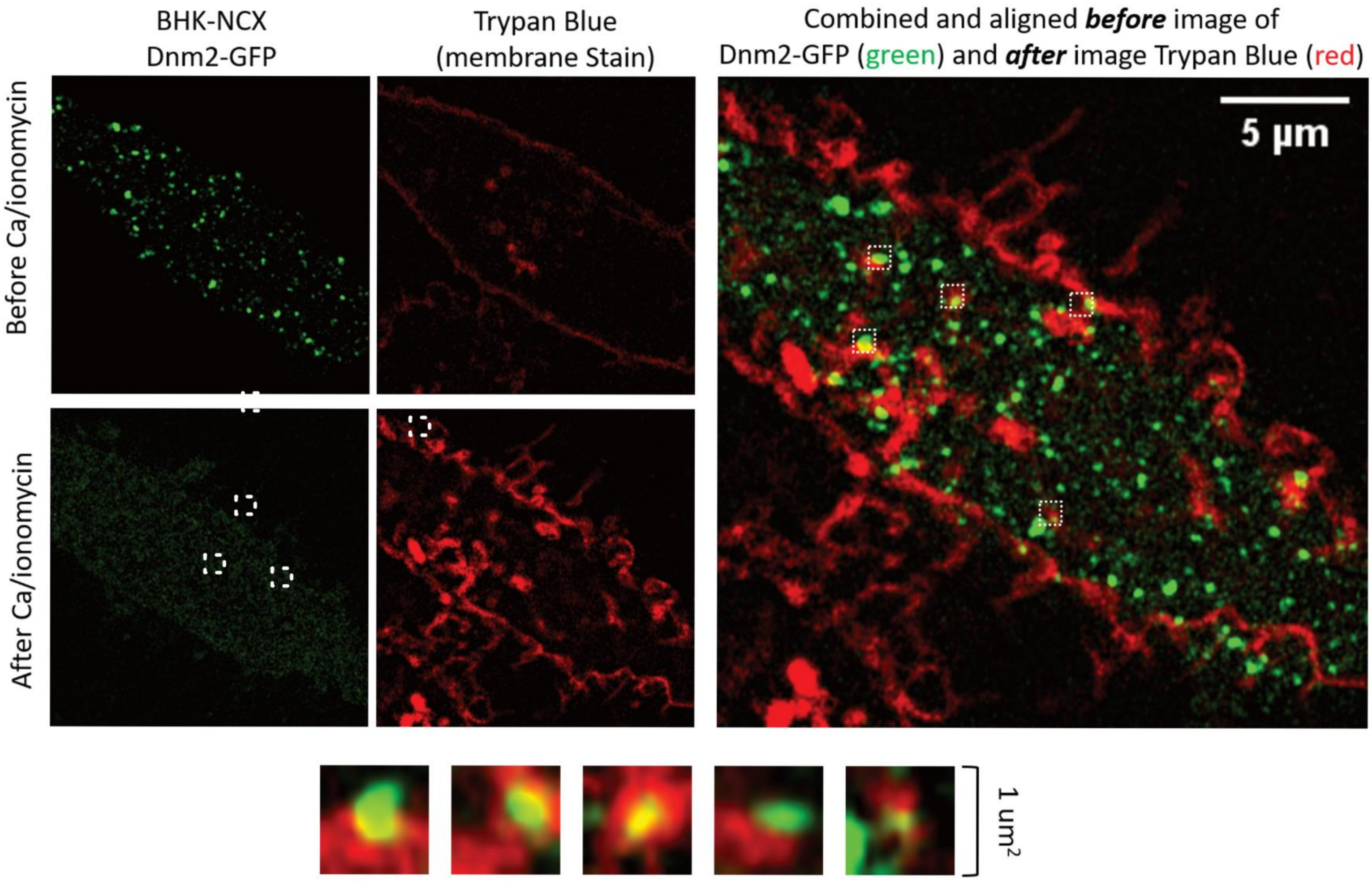

Supplemental Figure 6. BHK-NCX cells were transiently transfected with WT-Dnm2-GFP and live-cell imaged using super-resolution Airyscan mode. Dnm2-GFP punctae (green) are clearly visible at the membrane glass adhesion interface prior to Ca-ionomycin treatment. Concurrently, the membrane was visualized with 0.01% trypan blue.After Ca-ionomycin treatment, cells lose Dnm2 punctae as seen in MEF and HEK cells (see Fig. 1 and Sup. Fig. 3). Furthermore, trypan blue fluorescence increases with exposure of labeled membrane deeper within the cell (see Fig. 2). Alignment of the cell prior to Ca/ionomycin with trypan blue fluorescence after exposure reveals correlation of Dnm2-GFP punctae and increased TB labeling, n=3 independent cells from 3 experiments. Scale bar: 5 µm, inset 1 µm^2

Supplemental Figure 7.

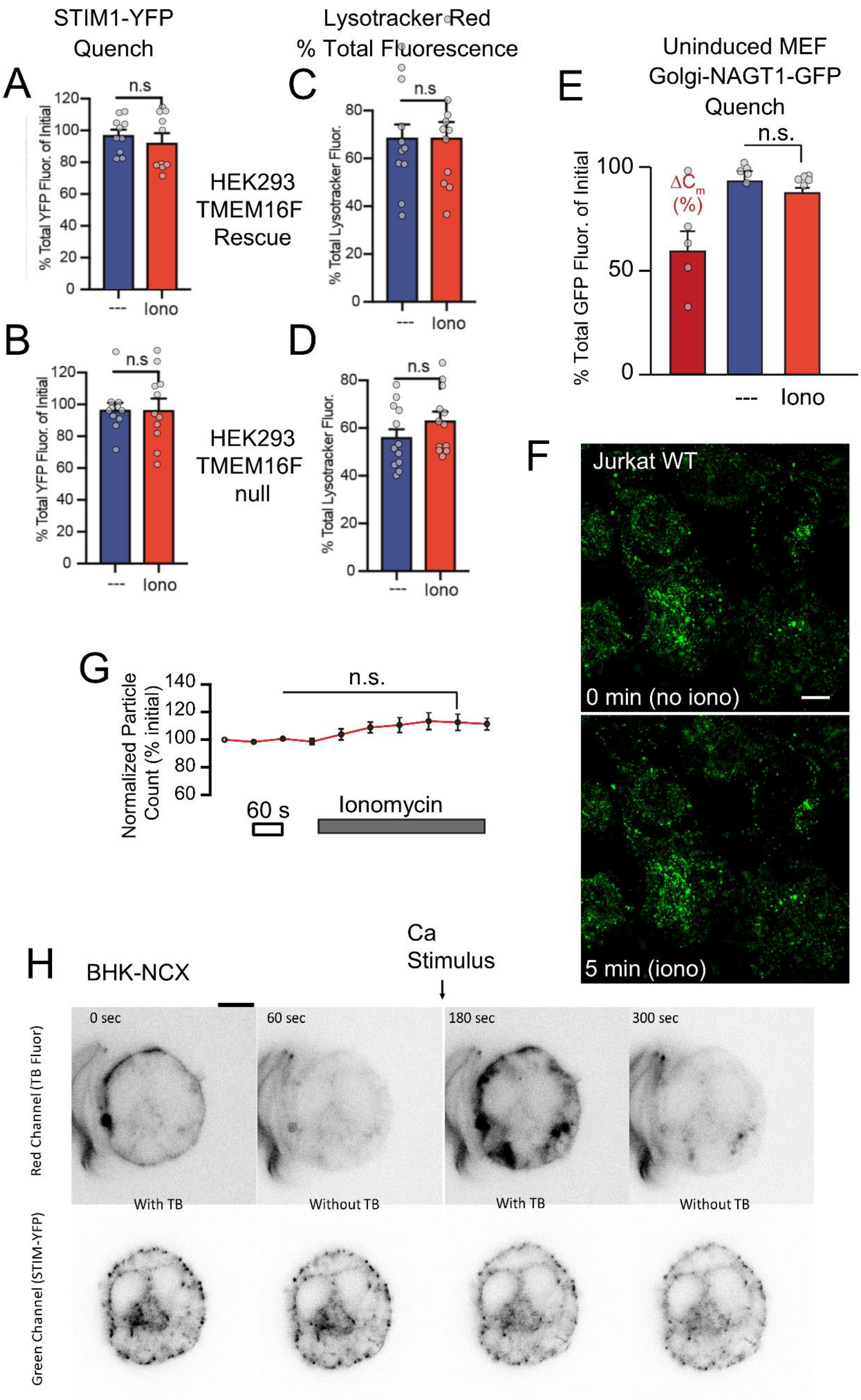

Supplemental Figure 7. Various organelle markers and recycled endosomes do not correlate with newly expanded PM (A-B) HEK-TMEM16F-null and murine TMEM16F-rescue cells transiently expressing the ER membrane protein STIM1 containing a luminal (ER resident) YFP fluorophore (Liou J. et. al., *Current Biology*, 2015) were imaged with extracellular transient application of 100 µg/ml TB quench extracellular exposure of YFP fluorescence. Cells were exposed to 5µM ionomycin to stimulate membrane fusion. Exposure of the luminal YFP domain of STIM1 during membrane expansion would allow for increased quenching of fluorescence by extracellular TB. (see H for representative images) No significant change in fluorescence was detected. Analysis shows percentage of initial YFP fluorescence after TB application (quench) prior to and 300 s after ionomycin stimulation. This represents the % of YFP lost during reversible quenching and therefore exposed STIM-YFP. For a representation of a positive quenching experiment see VAMP4 Sup. Fig. 8), n=5. Mean values and SEM depicted. Similar results also demonstrated no loss of exogenous KDEL-YFP, a luminal ER probe, after Ca-activated PM expansion, (see Sup. Vid. 6). (C-D) Newly expanded membrane is not associated with lysosomal probes. HEKTMEM16F-null and TMEM16F recue cells were stained for 60min with 100 nM of LysoTracker™ Red DND-99 (Molecular Probes). The probe is highly selective for acidic organelles and accumulates in lysosomal compartments. Cells were imaged before and after buffer was supplemented with 5µM ionomycin and 2 mM Ca. Lysosomal fusion to the PM would result in release of fluorescent cargo and loss of labelling. Analysis reveals no detectible loss of fluorescent punctae across both cell lines using ImageJ (NIH) to determine average fluorescence value per cell area with SEM before and after ionomycin stimulation, n=10. E) Golgi markers do not associate with newly expanded PM. The Golgi marker N-acetylglucosaminyl-transferase (NAGT1-GFP) where the luminal catalytic domain of NAGT1 was replaced with GFP (Nilsson, T. et. al., *JCB*, 1993) was expressed in uninduced Dnm TKO cells. After ionomycin treatment, C_m increased by an average of over 60% yet no change in observed quenching of luminal GFP (as in A-D) was observed, n=5. (F-G) Multi-Dimensional Super Resolution Airyscan Microscopy reveals no significant loss of recycled endosomes after membrane expansion. F) Representative micrographs illustrating FM-labelled punctae in WT Jurkat cells before and 5 min after ionomycin treatment. Jurkat WT cells were preincubated with FM4-64 for 1 hour to label endosomes, washed to remove extracellular dye, and attached to fibronectin coated imaging plates. Cell were washed with NaCl buffer and were supplemented with ionomycin after imaging was stabilized for a minimum of 4 frames. 30s frame times G) Quantification of projected 0.75 µm z-stacks reveals no significant change to the number of punctae before and after treatment. n=5 independent experiments. H) Representative micrographs of STIM-YFP (bottom) and Trypan Blue (Top) similar to (A-B). Calcium stimulus and PM expansion in BHK-NCX cells was activated at 180 sec using reverse NCX1. Reversible TB labeling clearly extends and washes from deep compartments while no correlation between ER-resident STIM-YFP is detected. No significant loss of YFP fluorescence is detected upon application of TB with only slow photobleaching of YFP, unrelated to TB application detected, n=3. All data was analyzed from the total number (n) of independent cells, or dishes for F-G, from a minimum of two experiments and expressed as mean ± s.e.m. Unpaired Student's *t*-test was used for comparing two groups. Scale bar: 5 µm

Supplemental Figure 8.

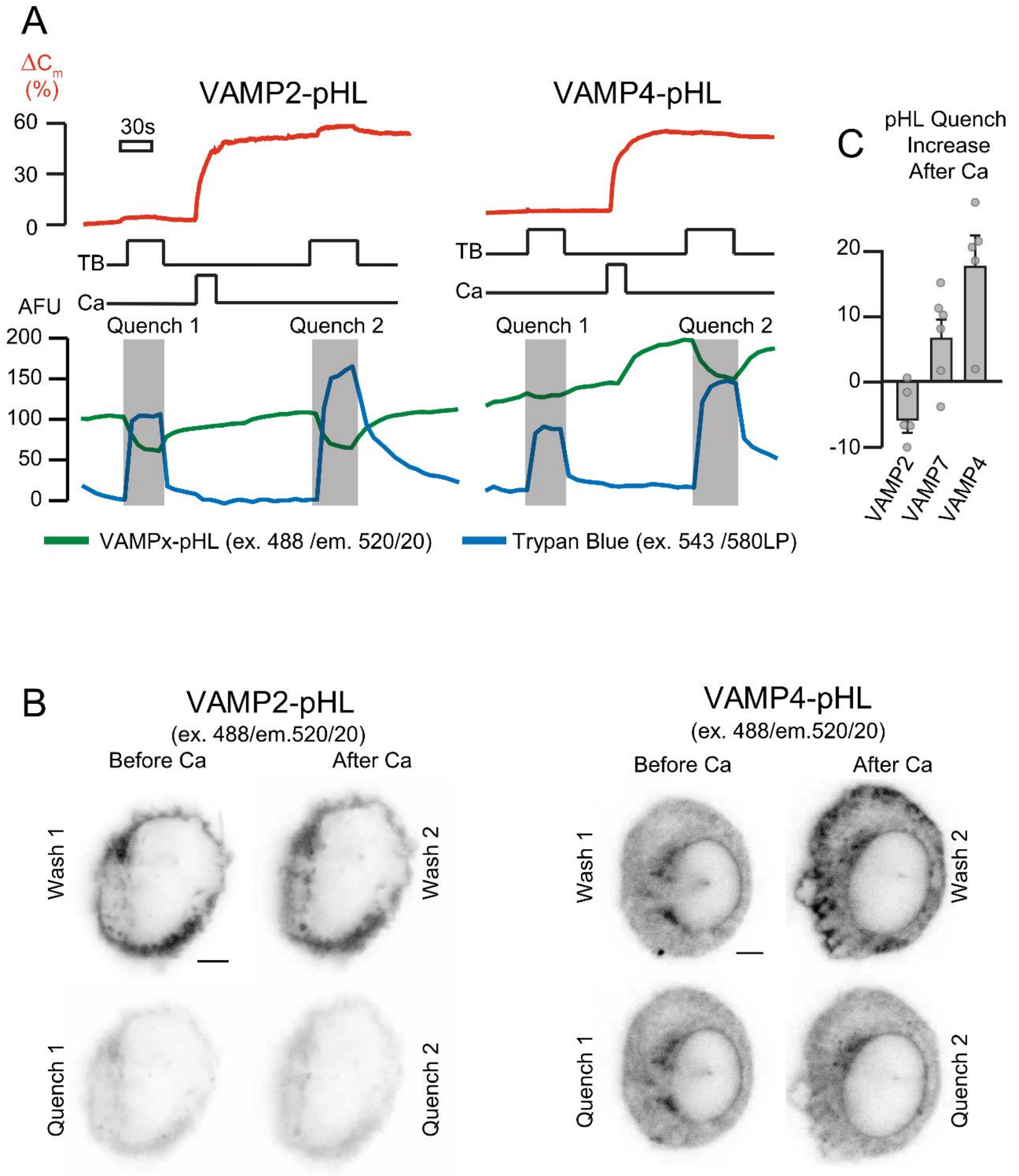

Supplemental Figure 8. Analysis of VAMP proteins in the PM during expansion. Among VAMP2, VAMP4- and VAMP7- pHlourin (pHl)fusion proteins, VAMP4 fusions preferentially increase cell surface expression after PM expansion. A) Representative time course of fluorescence and PM surface area changes B) Confocal images of BHK VAMP2-pHL (left) and VAMP4-pHL (right) before and after Ca-activated membrane expansion showing little increase in VAMP2 TB quenching and preferential increases in TB quenching of newly exposed VAMP4 fluorescence (see Sup. Vid. 7). C). Composite results indicate increase in VAMP quenching after Ca for VAMP2, 7, and 4. Values are the mean difference in quenching determined before and after PM expansion. As an illustration of TB quenching, prior to application of Ca, TB application on VAMP4 over-expressing cells (A, right) generated less than a 5% decrease in VAMP4-pHl fluorescence. After Ca, PM expanded over 50% and VAMP fluorescence increased as protein became exposed to the surface. When TB was applied again, VAMP fluorescence quenching increased to 26% of total signal. In VAMP2 expressing cells (A, left), quenching was observed at similar levels both before and after Ca elevations. PM area increased by a similar amount as seen in the VAMP4 cell. However, even with the increase in PM area, little increase in VAMP2 fluorescence and quenching was detected after Ca-elevation, n=5,6,5. Data was analyzed from the total number of independent cells (n) from a minimum of three experiments and expressed as mean ± s.e.m. Scale bar: 5 µm

Supplemental Figure 9.

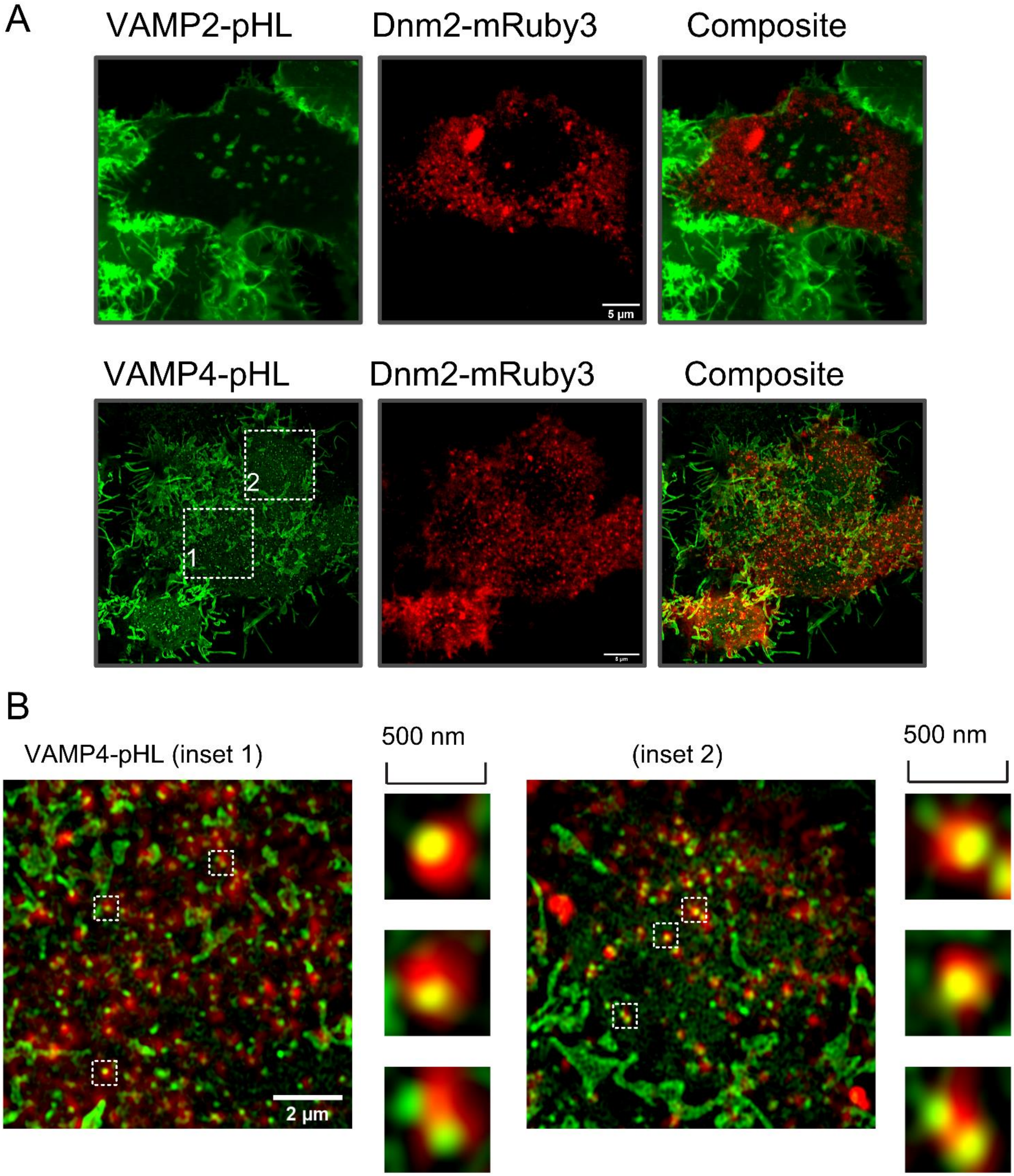

Supplemental Figure 9. VAMP4 but not VAMP2 correlates with Dnm2 punctae at the cell adhesion interface. A) Live cell super-resolution Airyscan images of HEK cells transiently co-expressing either VAMP2 (top, green) or VAMP4-pHlourin (bottom, green) with WT-Dnm2-mRuby3 (red). Dnm2-mRuby3 punctae are visualized near the cell adhesion interface with VAMP2 expression appearing primarily with surface membrane localization (see also Sup. Fig. 9) with minimal correlation between VAMP2 expression and Dnm2 expression. While VAMP4 does express at the PM surface, a significant number of punctae appear throughout the adhesion interface. These punctae correlate with expression of Dnm2-mRuby3 as detailed in below. Scale bar: 5 µm B) Inset 1 and 2 of VAMP4-pHL (green) and mRuby3 (red) with detailed 0.25 µm^2 pictographs highlighting consistent localization of VAMP4 with Dnm2 expression, n=3 (VAMP2), 4 (VAMP4). Scale bar (inset): 2 µm, pictograph: 0.25 µm^2.

Supplemental Figure 10.

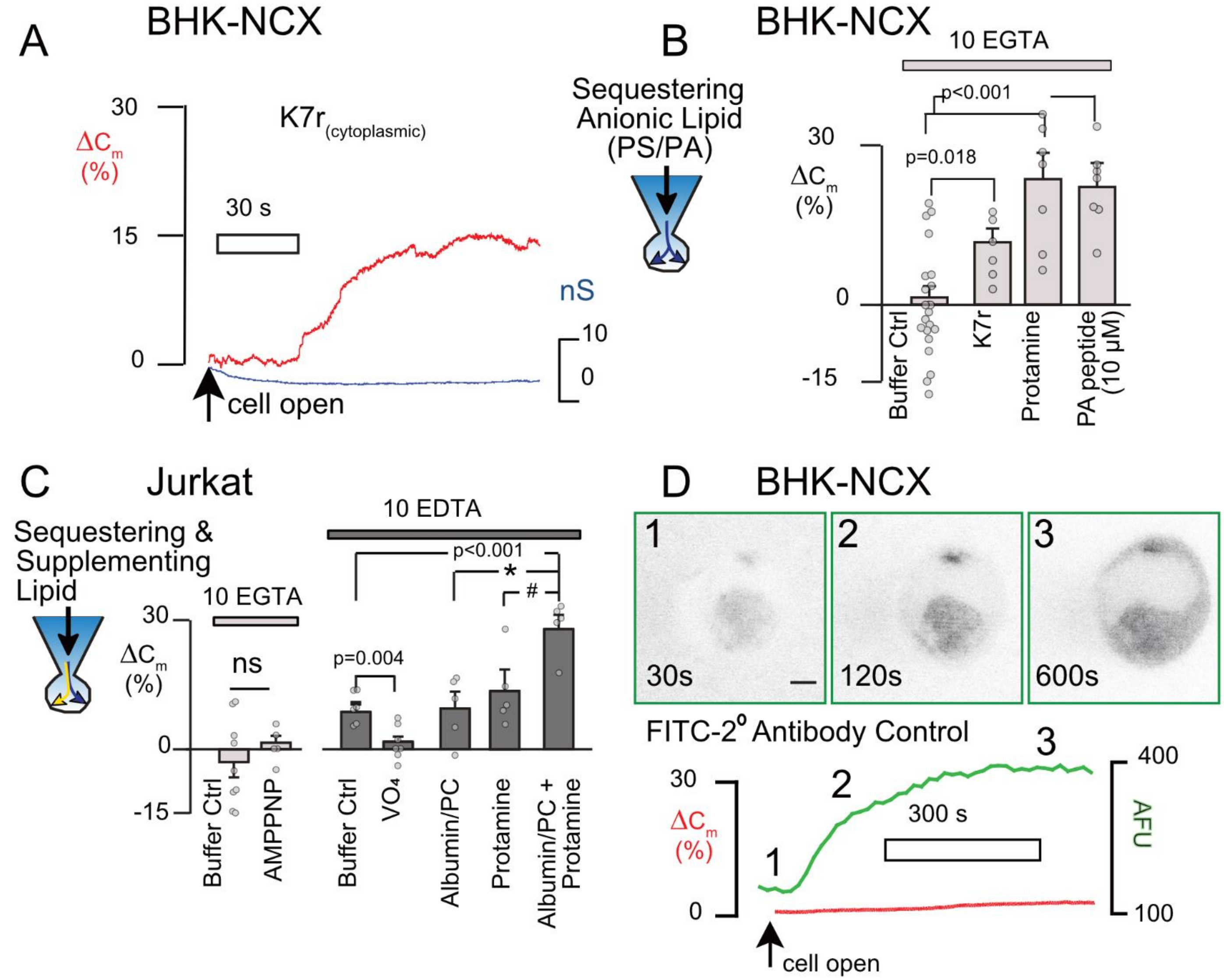

Supplemental Figure 10. As in Figure 3, manipulation of PLs and Dnm2 can expand PM compartments without Ca or TMEM16F activation. A) Sequestration of inner leaflet anionic PLs by cytoplasmic polycations and peptides in BHK cells. Slow modest expansion responses at room temperature in the absence of Ca reveal in BHK cell recordings with cytoplasmic K7r (100 µM) binding of anionic lipids in the inner leaflet sequesters free lipids eventually resulting in PM expansion. The slow process reveals the stochastic nature of compartment opening illustrated by C_m noise and steps as the new compartments are opened more slowly over time under these conditions. B) Composite results for BHK cells in similar conditions as Figure 3A with cytoplasmic dialysis of compounds that sequester anionic lipids from the inside. (0 ATP, 0 Ca, and 10 mM EGTA; K7r, 100 µM; protamine, 2 mg/ml; PA Peptide 10 µM), n=24,6,7,7. C) Cytoplasmic sequestration of anionic PLs and PC supplementation in Jurkat cells. No PM expansion detected during dialysis of 10mM cytoplasmic EGTA or EGTA with 4 mM AMPPNP. Expansion with 10 mM EDTA is blocked by VO_4 similar to MEF cells (Figure 3A). Sequestration of anionic PLs (protamine 2mg/mL) and PC supplementation (as in Fig. 3A for Dnm TKO cells) are not significant alone for Jurkat cells, but application of protamine with PC causes a synergistic 30% expansion response (*p=0.003; $^{\#}$p=0.013), n=9,5,6,7,5,5,5. D) Figure 3B control conditions. C_m recordings of BHK cells (red) in Ca-free (10 mM EGTA) conditions for over 10 min during cytoplasmic dialysis with FITC-labelled goat secondary antibody. PM expansion does not occur under these conditions and cytoplasmic fluorescence (green) shows proper dialysis of antibodies with a half-maximal response in ~3 min (n=5). Live-cell pictographs (right) reveal the extent of cytoplasmic diffusion of secondary antibody while no change in PM area is detected. All data analyzed from the total number of independent cells (n) from a minimum of two experiments and expressed as mean ± s.e.m. Unpaired Student's *t*-test was used for comparing two groups. Scale bar: 5 µm
