# Supplementary material for: TMEM16F and dynamins control expansive plasma membrane reservoirs
Source: Nat Commun. 2021 Aug 17;12:4990. doi: 10.1038/s41467-021-25286-z (PMC8371123; doi:10.1038/s41467-021-25286-z)
Supplement: Supplementary file 3 — Description of Additional Supplementary Files [file 41467_2021_25286_MOESM3_ESM.pdf]

Description of Additional Supplementary Files

File Name: Supplementary Movie 1

Description: Jurkat T cells were loaded with the cytoplasmic calcium indicator Fluo4-AM (3 µM, green) for 30 min at RT, media was replaced with isotonic 140 mM NaCl based standard extracellular solution. Cells were imaged at 37° C using confocal microscopy with a 40x objective and then treated with 5 µM ionomycin for 400s in the presence of polylysine-rhodamine (K7r) (red) which binds rapidly (within seconds) to exposed phosphatidylserine. Confocal microscope images of Fluo4-AM and K7r fluorescence in a single field of cells are shown (scale bar is 10µm). Increases in intracellular Ca (green) are clearly visible prior to extracellular binding of K7r to anionic PLs (red). Subsequent to PM expansion an PL scrambling, large scale membrane shedding can be seen when exposure to ionomycin is prolonged. While shedding exists for multiple cell types, shorter or transient increase in Ca reduce the amount of shedding. For more details into ionomycin and Ca induced exosomal release and shedding, see our previously published paper (Bricogne et. al., *Scientific Reports*, 2019).

File Name: Supplementary Movie 2

Description: Video Representation of Supplemental Figure 5 detailing the reversibility of K7r binding into discrete sub-compartments or invaginations of the PM. Reversible labeling of punctae is due to wash on and off of extracellular K7r as described in Sup. Fig. 5. In the presence of K7r and due to the slower activation of TMEM16F and PM expansion (4 µM free cytoplasmic Ca), over longer periods of time (min 6-9) localized punctae dissipate as anionic lipids diffuse and laterally distribute in the PM.

File Name: Supplementary Movie 3

Description: Video Representation of Figure 2C detailing the reversibility of TB binding into discrete sub-compartments or invaginations of the PM.

File Name: Supplementary Movie 4-5

Description: 5 Z-stack representation (0.25 µm/step) of BHK cells fixed for 10 min at RT using 2.5% EM grade glutaraldehyde in 0.2 M Na cacodylate (pH 7.4). BHK-NCX cells were grown on glass bottom dishes (MatTek) in standard DMEM media. Cell culture media was replaced with 120 mM NMDG, 15 mM Hepes, 15 mM TEAOH, 125 mM Aspartic Acid, 2 mM $MgCl_2$ and 0.5 mM EGTA buffer to minimize NCX mediated efflux of cytoplasmic Ca. Buffer warmed to 37°C containing in addition Ca (2 mM) alone (Sup. Vid. 4) or Ca plus ionomycin (5 µM) (Sup.Vid. 5) was added for 180 sec and washed 3 times with RT NMDG buffer containing no Ca. TB (100 µg/ml) was added to the cells post fixation and confocal imaging at 63x was performed on a Zeiss LSM 880 at 512 x 512 with 32 detector Airyscan and post-processing. Excitation: 561 nm; Emission: 580LP. Inverted high-resolution imaging details that TB labeling did not permeate the membrane and fixed cells were not permeabilized. Permeabilized cells are readily visualized and excluded due to extensive labelling of the nucleus and cytoplasm. For cells containing Ca alone (Sup. Vid. 4) minimal labeling of invaginated compartments were detected. For cells containing ionomycin plus Ca a clear increase in tubulated compartments can be seen. As the TB can only label exposed membrane and was applied after fixation these compartments must remain exposed to the extracellular space and do not represent isolated endocytic compartments. Visualization of the necks and constrictions at the surface of the PM detail the extent of these newly exposed membrane compartments.
