# Supplementary material for: TMEM16F and dynamins control expansive plasma membrane reservoirs
Source: Nat Commun. 2021 Aug 17;12:4990. doi: 10.1038/s41467-021-25286-z (PMC8371123; doi:10.1038/s41467-021-25286-z)
Supplement: Supplementary file 11 — Reporting Summary [file 41467_2021_25286_MOESM11_ESM.pdf]

nature portfolio

Corresponding author(s): Michael Fine

Last updated by author(s): Jul 1, 2021

Reporting Summary

Nature Portfolio wishes to improve the reproducibility of the work that we publish. This form provides structure for consistency and transparency in reporting. For further information on Nature Portfolio policies, see our Editorial Policies and the Editorial Policy Checklist.

Statistics

For all statistical analyses, confirm that the following items are present in the figure legend, table legend, main text, or Methods section.

n/a	Confirmed	
☐	☒	The exact sample size (*n*) for each experimental group/condition, given as a discrete number and unit of measurement
☐	☒	A statement on whether measurements were taken from distinct samples or whether the same sample was measured repeatedly
☐	☒	The statistical test(s) used AND whether they are one- or two-sided<br>*Only common tests should be described solely by name; describe more complex techniques in the Methods section.*
☒	☐	A description of all covariates tested
☐	☒	A description of any assumptions or corrections, such as tests of normality and adjustment for multiple comparisons
☐	☒	A full description of the statistical parameters including central tendency (e.g. means) or other basic estimates (e.g. regression coefficient) AND variation (e.g. standard deviation) or associated estimates of uncertainty (e.g. confidence intervals)
☐	☒	For null hypothesis testing, the test statistic (e.g. *F*, *t*, *r*) with confidence intervals, effect sizes, degrees of freedom and *P* value noted<br>*Give P values as exact values whenever suitable.*
☒	☐	For Bayesian analysis, information on the choice of priors and Markov chain Monte Carlo settings
☒	☐	For hierarchical and complex designs, identification of the appropriate level for tests and full reporting of outcomes
☐	☒	Estimates of effect sizes (e.g. Cohen's *d*, Pearson's *r*), indicating how they were calculated

Our web collection on statistics for biologists contains articles on many of the points above.

Software and code

Policy information about availability of computer code

Data collection

For data collection a custom Matlab (v2015) code (Capmeter V7) was used to collect electrophysiological data with references provided. The code and software is publicly available and cited. (https://sites.google.com/site/capmeter/) See JGP Wang and Hilgemann (2008) 132 (1):51-65 for detailed information. For optical data as indicated in Methods, Nikon EZC1 software (v3.9) was used to capture confocal imaging and Zeiss Blue (v2.1) LSM 880 was used for super-resolution airyscan images. For AnnexinV staining Aurox Clarity system with Vision software was used for data collection and analyzed using Fiji (ImageJ 1.52p, NIH)

Data analysis

For data analysis, Excel v2105 (Microsoft) was used for basic data analysis (mean, SD, SEM) and final analysis, error, normality, student t-tests (two sided, paired or unpaired as indicated) and figure creation was performed using SigmaPlot v14.5 (SyStat) for composite analysis. ImageJ (1.52p, NIH) was used for image analysis and video rendering.

For manuscripts utilizing custom algorithms or software that are central to the research but not yet described in published literature, software must be made available to editors and reviewers. We strongly encourage code deposition in a community repository (e.g. GitHub). See the Nature Portfolio guidelines for submitting code & software for further information.

Data

Policy information about availability of data

All manuscripts must include a data availability statement. This statement should provide the following information, where applicable:

- Accession codes, unique identifiers, or web links for publicly available datasets
- A description of any restrictions on data availability
- For clinical datasets or third party data, please ensure that the statement adheres to our policy

The authors declare that all data supporting the findings of this study are available within the article and its Supplementary Data files, and available from the

corresponding authors and Prof. Donald Hilgemann on request. There are no restrictions on data availability. In addition, Source data are provided with this paper with individual Figure File raw data included.

Field-specific reporting

Please select the one below that is the best fit for your research. If you are not sure, read the appropriate sections before making your selection.

☒ Life sciences ☐ Behavioural & social sciences ☐ Ecological, evolutionary & environmental sciences

For a reference copy of the document with all sections, see nature.com/documents/nr-reporting-summary-flat.pdf

Life sciences study design

All studies must disclose on these points even when the disclosure is negative.

Sample size	No statistical method was used to predetermine sample size. Sample size chosen for statistical analysis is typical for electrophysiological analysis as previously published and statistical power (normality) was determined using the Shapiro-Wilk test on Sigmaplot (Systat) Individual cellular recordings are considered independent experiments with replicates of experimentation repeated a minimum of two times on multiple days with a minimum data set of n=4. See Kang and Hilgemann, Nature, 427, 544-548 (2004)
Data exclusions	In general, no data was excluded. On large data sets (Fig. 1C (bottom); n>10) statistical outliers may be considered as single data points greater that two standard deviations from the mean as stated in Methods.
Replication	Data replication was performed for all statistical experiments with data acquisition performed on multiple systems and independent experiments. If replication was not successful, data was not considered valid. For combined confocal and electrophysiology data acquisition was only available on one system and was replicated a minimum of two separate experiments.
Randomization	Allocation of samples into experimental groups is not relevant to the study of individual cellular membrane capacitance as, unless otherwise stated, all cell treatments start and end within the same cell and outcomes are measured on an individual cell basis.
Blinding	As described above, blinding is not relevant to this study. Typical experiments involve an individual cell with its own baseline and treatment condition. For experiments involving membrane diameter and capacitance (Fold Excess Membrane (Fig. 1C) blinding was performed and cell induced, non-induced or expressing K44A dominant negative of K562E lipid binding mutant dynamin or mCherry control were blinded to the electrophysiologist to improve the reliability and significance of the data.

Reporting for specific materials, systems and methods

We require information from authors about some types of materials, experimental systems and methods used in many studies. Here, indicate whether each material, system or method listed is relevant to your study. If you are not sure if a list item applies to your research, read the appropriate section before selecting a response.

Materials & experimental systems

n/a	Involved in the study	
☐	☒	Antibodies
☐	☒	Eukaryotic cell lines
☒	☐	Palaeontology and archaeology
☒	☐	Animals and other organisms
☒	☐	Human research participants
☒	☐	Clinical data
☒	☐	Dual use research of concern

Methods

n/a	Involved in the study	
☒	☐	ChIP-seq
☒	☐	Flow cytometry
☒	☐	MRI-based neuroimaging

Antibodies

Antibodies used	Antibodies used were from Abcam (Dnm1 [EP772Y], Ab52852; Dnm2, Ab3457; Brain Naturietic peptide, Ab19645). Secondary IgG fluorescent control was goat anti-guinea pig Alex Fluor-488 (Invitrogen, A11073). Dnyab was a gift from Aurelien Roux, Department of Biochemistry, University of Geneva, Geneva, Switzerland, please see Galli et. al., eLife, 2017; 6, e25197
Validation	Ab52852 is rabbit monoclonal and is knockout validated on human U2OS cells and commonly used for WB/IP for murine and human use on Abcam website. Ab3457 is a rabbit polyclonal with validated reactivity to murine, rat, human and primates and is available on Abcam's website. ab19645 is rabbit polyclonal corresponding to SPTPQRRPVSSIHPPGRPPA and is validated for rat human mouse and primate. It is routinely used for ICC, WB IHC and is available through Abcam's website, no other validation stated. Similarly, BNP is a rabbit polyclonal used as a negative control validated using WB and IF, no other documentation stated. For Dynab validation please see Galli et. al., eLife, 2017; 6, e25197 received as a gift directly from the supplier - no additional validation performed.

Eukaryotic cell lines

Policy information about cell lines

Cell line source(s)	HEK293T (ATCC, CRL-11268), HEK-TMEM16F-null (ECACC, modified at UCL (See Bricogne, 2019 ref), Jurkat E6.1 T cells (ECACC, 88042803) and Jurkat TMEM16F-null (ECACC, modified - See Bricogne, 2019); MEF-Dnm TKO cells (Pietro De Camilli, Yale), H1299 cells (modified by S. Schmid, UTSW, ATCC ,CRL-5803), Neuro2A (Ege Kavalali, U Vanderbilt) BHK-NCX cells (gift from Ken Philipson and Hilgemann lab, See Linck et. al., 1998)
Authentication	For cells purchased from ATCC or ECACC, authentication was performed by supplier using STR (short tandem repeat) Profiling Authentication analysis. For gifts and modified cell lines from external sources (MEF Dnm TKO, H1299 Dnm 1 KO, Neuro2A, BHK-NCX) no genetic authentication was performed however the original source is provided within the Methods. No in-house genetic authentication was performed.
Mycoplasma contamination	All cell lines are routinely monitored using PCR based mycoplasm tests (ATCC) and treated with Plasmocin (Invivogen) or discarded if contamination is detected. No data included the paper results from mycoplasm infected cells. Primary cardiac mycoytes used in supplemental figure 1 are not cultured and do not undergo mycoplasm testing but no statistical data is included on these cells for this paper.
Commonly misidentified lines (See ICLAC register)	N/A No commonly misidentified lines utilized on the ICLAC as of June, 2021
